# Effect of berry-based supplements and foods on cognitive function: a systematic review

**DOI:** 10.1038/s41598-022-07302-4

**Published:** 2022-02-25

**Authors:** Negar Bonyadi, Neda Dolatkhah, Yaghoub Salekzamani, Maryam Hashemian

**Affiliations:** 1 Student Research Committee, Tabriz University of Medical Sciences, Tabriz, Iran; 2 Physical Medicine and Rehabilitation Research Center, Aging Research Institute, Tabriz University of Medical Sciences, Tabriz, Iran; 3 Department of Biology, School of Arts and Sciences, Utica College, Utica, USA

**Keywords:** Diseases, Medical research, Neurology

## Abstract

In the current decade, a growing body of evidence has proposed the correlation between diet and cognitive function or dementia in the ageing population. This study was designed to appraise discoveries from the randomized controlled trials to confirm the effects of berry-based supplements or foods on cognitive function in older adults. PubMed/MEDLINE, Cochrane Central Register of Controlled Trials, Web of Science, Scopus, EMBASE, Google Scholar, and ProQuest as well as SID, Magiran, and Iranmedex electronic databases were explored for human interventional studies up to March 2021. In total, eleven articles were identified using frozen blueberry (n = 4 studies), blueberry concentrate (n = 2), beverage (n = 3), capsule (n = 1), extract and powder (n = 1). These studies had been performed among older people with no recognized cognitive impairment or mild cognitive impairment (MCI). The primary outcomes included global cognitive function, psychomotor function, learning and memory, working memory capacity, executive functions, and brain perfusion/activity. To our knowledge, this is the first systematic review of available clinical trials on the effects of berry-based supplements and foods on cognitive performances as well as brain perfusion parameters among the elderly with normal cognition or MCI. Existing evidence concludes that berry-based supplements and foods have beneficial effects on resting brain perfusion, cognitive function, memory performance, executive functioning, processing speed, and attention indices.

## Introduction

Along with the aging of the population worldwide, cognition-related diseases are progressively rising^1^. These disorders, such as mild cognitive impairment (MCI), dementia, and Alzheimer's disease (AD) significantly increase the burden of social and economic health for most communities^2^. The total numeral of individuals with dementia is almost 35.6 million as declared by world health organization (WHO), and by 2050 the total predictable prevalence of AD is anticipated to be 135.5 million^3,4^.

Numerous studies have shown a relationship between lifestyle influences and cognitive function in older adults^5–7^. A considerable amount of data has specified that nutrition is related to age-associated disorders and longevity^8,9^. It has been shown that certain healthy dietary patterns, foods, and micronutrients, such as Mediterranean diet^10^, vitamin C^11^, vitamin E^12^, omega-3 fatty acids^13^, polyphenols, including flavonoids^14^, fruits and vegetables^15^, tea^16^, coffee^17^ and milk^18^, have protective effects on cognitive disorders and dementia.

An initial causal factor to both normal and pathological alterations in brain operation is the agglomeration of oxidative impairment along with diminished endogenous antioxidant barricades^19,20^. The brain is principally exposed to oxidative injury because of its high amount of oxygen intake^21^. Vegetal foods, comprising vegetables, fruits, as well as their juices, are the main human origins of exogenous anti-inflammatory and antioxidant compounds. Additionally, flavonoids are very effective in preserving neurons, improving remaining neuronal operation, increasing neuronal restoration, and inducing neurogenesis^22,23^. Particularly, flavonoids may raise the quantity of between-neuron connections as well as its strength, through their interactions with critical neuronal intracellular signaling paths, which are crucial in adjusting the survivance, differentiation, and long-term potentiation of neurons, and also memory^24^. Though the exact site of flavonoids interaction with signaling paths is uncertain, it seems that they exert their action by connecting to adenosine triphosphate (ATP) locations on receptors and enzymes, modifying the kinase activities directly, affecting the acting of vital phosphatases, functioning in antagonism to kinases, stabilizing Ca^2+^ homeostasis in neurons, and affecting signaling pathways lying downstream of kinases^25,26^.

Preliminary animal studies have established that antioxidant-rich dietary patterns could postpone and even reverse age-associated cognitive decline in laboratory animals^27^. Polyphenols are among the most plentiful antioxidants in the human diet^28^. Recent efforts have proven that berry supplementation (i.e. blueberries and strawberries) can encourage dramatic alterations in the brains of animals besides their currently well-known antioxidant properties^29,30^. The chemical composition of berries is changeable based on the developing locality and ecological environments, plant nourishment, maturity phase, and harvest time, as well as later storage conditions^31^. Main types of polyphenolic flavonoids in berries consist of flavan-3-ols, flavanols, and anthocyanidins^32^. Along with anthocyanins being the principal flavonoids in berries, flavan-3-ols and flavonols are also present in lesser amounts^33^. The health benefits of anthocyanins have been broadly defined, particularly in the prevention of oxidative stress related disorders, such as neurodegenerative diseases^34^. Additionally, current evidence proposes that health-promoting properties of anthocyanins may also be correlated to modification of gut microbiota^35^.

There is some evidence suggesting that polyphenols from berries may be presenting a multiplicity of impacts on the aging brain comprising antioxidant properties, vascular consequences, gluco-regulation, neuro-synthesis, and gut microbiota modifications^25,36–41^. Berry fruits and their chemical ingredients facilitate signaling pathways including those involved in cell longevity as well as increasing neuroplasticity, neurotransmission, and neuronal calcium homeostasis, all of which reduce the age-related decline in behavior^40^. Berry supplementation of animals with damaged hippocampus not only improved the number of neurons surviving but also decreased the amount of stimulated microglia and reduced the pro-inflammatory cytokine expressions^42^. Additionally, berry supplementation was correlated with an improved capability of neurons to preserve learning-related physical and practical adjustments in the hippocampus. Berry supplementation was revealed to persuade stimulation of a multifunctional transcription factor most especially raised throughout the consolidation of short-term to long-term memory in the hippocampus. This activation was related to an improved expression of the growth factor brain-derived neurotrophic factor (BDNF), which has been widely involved in the preservation of neuronal function through aging. Furthermore, dietary blueberry supplementation was revealed to modify the in vivo expression and activity of stress and survival-correlated signaling molecules^43^. Normally consumed berries such as blueberries and strawberries also show anti-glycative activity.^44^ A two-percent blueberry diet intake for four months improved appreciation of novelty in old rats^45^. Additionally, a 2-percent blueberry diet improved motor function in old animals and led to enhanced balance plus coordination^46^. However, the available indications for strawberry’ improvement of motor performance in old mice and rats are less obvious^46–49^.

Many recent clinical studies have explored the antioxidant and cognition-sparing properties of berries in humans^37,50–52^. Numerous clinical trials have been conducted on the effects of berry-based foods or supplements on cognitive function and memory. Elderly with MCI who consumed blueberry juice (6–9 mL/kg/day) for three months exhibited improved late recall for word lists in the California Verbal Learning Test (CVLT), as compared to the baseline, a tendency to improved functioning, compared to placebo controls and better act in the Verbal Paired Acquaintances Learning Test relative to both baseline and placebo controls^53^.

Due to the lack of systematic review in this regard, the present review study was planned to review recent indications for the beneficial properties of berry-based supplements and foods on cognitive components in the elderly and middle-aged healthy subjects or subjects with MCI.

## Methods

The main objective of the current systematic review was to evaluate the efficacy of whole berries or a berry-based products (e.g. smoothie, juice) or berry extract/capsule consumption in adult or old subjects with healthy cognitions or MCI. The present study has been prearranged according to the guidelines and checklist in Cochrane Handbook for Systematic Reviews of Interventions (version 5.1.0)^54^ and “Preferred Reporting Items for Systematic Reviews and Meta-Analyses (PRISMA)” statement^55,56^. The investigation question was structured based on the PICOS (participants, interventions, comparators, outcomes, and study design) criteria (Table 1) as follows: Do berry-based dietary supplements or foods affect at least one recognized cognition related outcome in adult or old subjects with healthy cognitions or MCI?^57^.

**Table 1:** PICOS criteria for inclusion and exclusion of studies.

Parameter	Description
Population	Adult or old subjects with healthy cognition or mild cognitive impairment
Intervention	Whole Berries/Berry-based Food/Berry Extract/Berry Supplement
Comparator	Any comparator
Outcomes	Outcomes regarding at least one of the following indices: episodic memory, long-term memory, short-term memory, working memory, executive function, psychomotor reaction time, attention
Study design	Randomized controlled clinical trial with a crossover or parallel design

### Literature search

The online databases (MEDLINE, Web of Science, Cochrane Library, Scopus, EMBASE, Google Scholar, Clininaltrial.gov, Science direct, and ProQuest in addition to SID, Magiran, Irandoc, and Iranmedex for Persian language literature) were employed for search across the titles, abstracts, and keywords of all articles for eligibility up to March 2021. Duplicate studies were excluded. Further, a manual search of the references of the reviewed studies was applied as additional resources to identify other suitable articles that were missed by the electronic search.

A web-based systematic literature exploration was executed by means of the subsequent MeSH terms: (“randomized controlled trial” OR “RCT” OR “controlled trial” OR “intervention trial” OR “intervention study”) AND (Chokeberry OR “Chokeberry extract” OR blackberry OR “blackberry extract” OR raspberry OR “raspberry extract” OR blueberry OR “blueberry extract” OR strawberry OR “strawberry extract” OR cranberry OR “cranberry extract” OR cranberry OR “cranberry extract”) AND (“memory”(all Fields) OR “cognitive”(all Fields) OR “cognition”(all Fields) OR “forgetfulness”(all Fields) AND (“older”(all Fields) OR “adult”(All Fields) OR “elderly”(all Fields) OR “older”(all Fields) NOT (animal OR mouse OR mice OR rat OR pig OR cell OR in vitro OR systematic review)). After the primary search, titles and abstracts were sent out from EndNote X8 into Microsoft Excel to be screened. All saved articles were evaluated independently by two investigators (NB and ND) regarding titles and abstracts. Any differences were considered and resolved through consensus or by a third independent investigator (YS).

### Inclusion and exclusion criteria

Table 1 reports the PICOS (Participant, Intervention, Comparators, Outcomes, and Study design) criteria accepted in this systematic review of clinical trials. The articles were limited to those published in English or Persian. Inclusion criteria were interventional studies implemented on humans examining the effects of whole berries or a berry-based products (e.g. smoothie, juice) or a berry extract/capsule supplementation on the defined cognitive-related outcomes. The outcomes included global cognitive function, psychomotor function, learning and memory, working memory capacity, executive functions, and brain perfusion/activity in adults and elderlies appraised to have healthy cognitions or MCI. Studies were also excluded if the participants had dementia; if the outcome of the study was not cognitive-related or if the study design was review article, semi- empirical study without a control arm, animal study, trial protocol, letter to the editor, case report, case series, observational study (cross-sectional, case–control and cohort), and unpublished trials.

### Data collection

The information of studies regarding the year of publication, participant characteristics, demographic details, location, study design/methodology, interventions (protocol and duration), sample size, dropout and primary outcomes were extracted for each included study by the investigators (NB, ND and YS).

### Quality assessment

To assess the risk of systematic errors in the involved trials fulfilling the eligibility criteria, two authors (ND and MH) independently estimated the potential risk of bias based on the Cochrane Collaboration’s tool for evaluating the risk of bias^58^. Briefly, the trial quality principles consisted of evaluation of: "randomization sequence generation, outcome assessment, blinding of subjects, personal and allocation concealment, imperfect outcome data, and discerning outcome reporting, as well as other sources of bias". Any discrepancies were debated and resolved by agreement or by a third independent reviewer (YS) if required. All trials were determined for each series of bias separately, and the trials were considered to have a score of bias as “low risk,” “high risk,” or “unclear risk” if the information was insufficient.

### The outcome measures

The review's primary outcomes included episodic memory, long-term, and short-term memory, working memory, executive function, psychomotor reaction time, and attention.

## Results

### Study selection process

**Figure 1:**
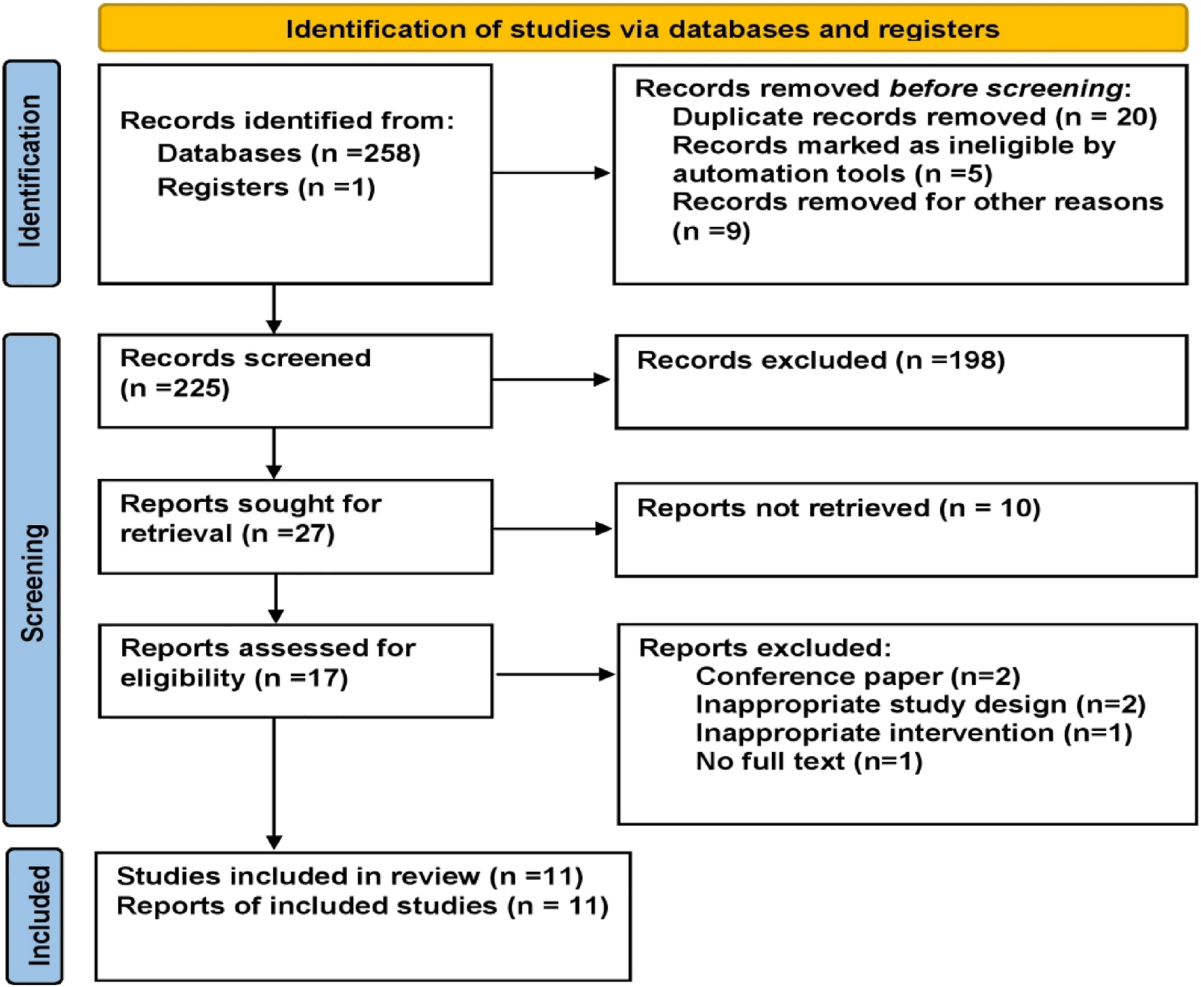
PRISMA flow chart summary of the systematic review search process.

**Figure 2:**
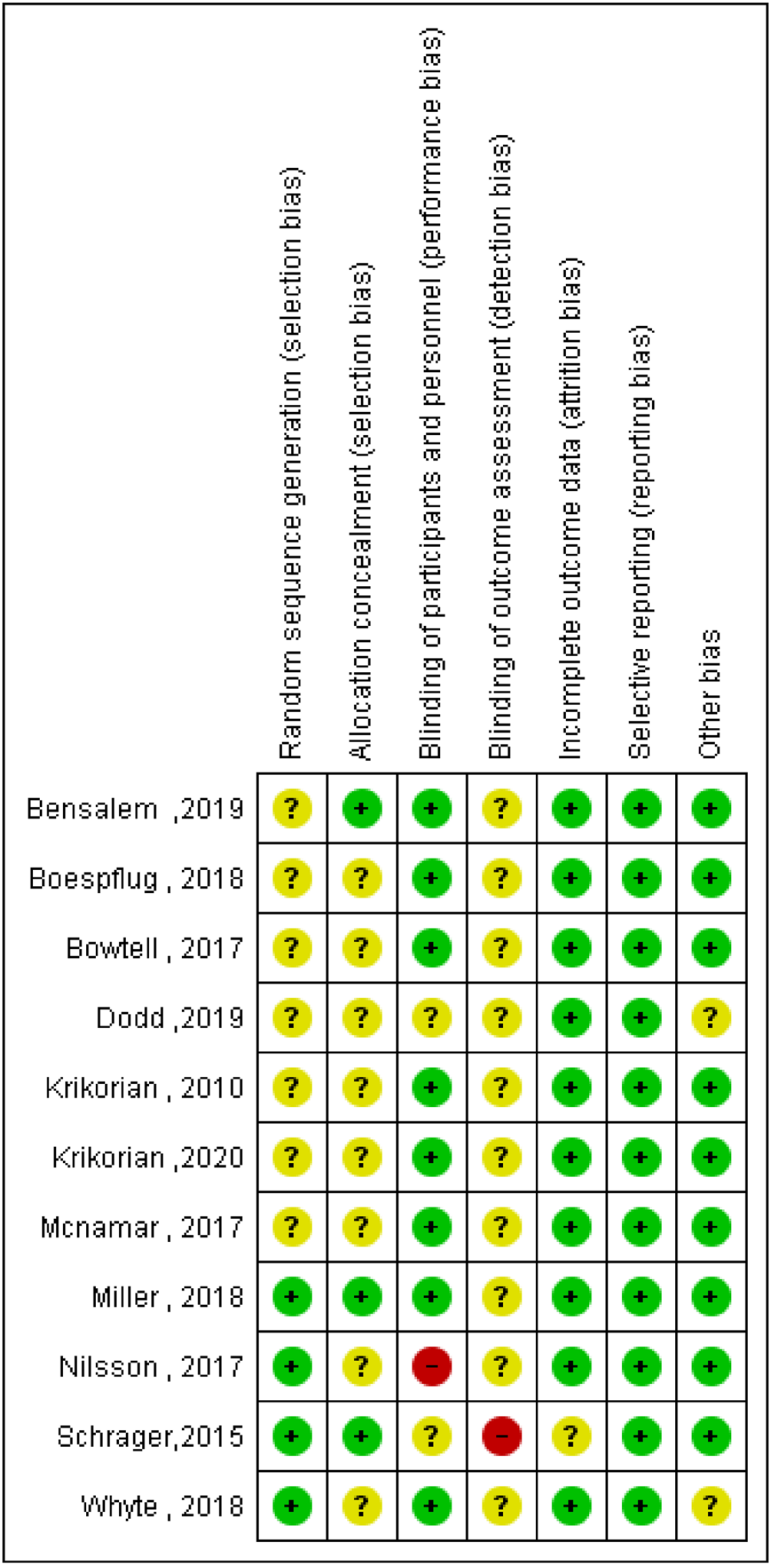
Risk of bias.

A flowchart explaining the study selection steps is presented in Fig. 1. A total of 259 studies were recognized from the primary database searches, and after eliminating the duplicates, 225 studies remained. Of these, 208 were disqualified after screening the title and abstracts of studies. The remaining 17 studies were evaluated in full text whereby 11 studies fulfilled the eligibility criteria (Fig. 1). The results of quality assessment of included studies is presented as Fig. 2.

### Study characteristics

Of the included studies, seven were randomized parallel group (n = 7), two were crossover, and one was a pilot study (n = 1) (Table 2).

**Table 2:** Included randomized controlled trials of berry-based food interventions on cognitive function.

Author (year)	Location	Inclusion criteria	Sample size and treatment (dosage)	Sample size at the end of treatment	Design and study duration	Main outcomes	Main results
Krikorian et al.^53^	USA	Age > 65 years, with age-related mild, acquired memory decline corresponding to MCI	Wild BB juice 6–9 mL/kg/day (444 mL/day in individuals weighing 54–64 kg, 532 mL/day in individuals weighing 65–76 kg, and 621 mL/day in individuals weighing 77–91 kg) (n = 9)	–	Pilot, single-blind?, one-arm trial, 12 weeks	Verbal learning, including V-PAL and CVLT	Improved V-PAL (*p* = 0.009), and CVLT (*p* = 0.04) following supplementation
Schrager et al.^62^	USA	Healthy older adults and age > 60 years old	1. Flash-frozen BB (2 cups/day) (n = 13) 2. Placebo/carrotjuice drink (n = 7)	1. Flash-frozen BB (2 cups/day) (n = 13) 2. Placebo/carrotjuice drink (n = 7)	Parallel group, open-label RCT, 6 weeks	Psychomotor/adaptive gait function and executive function (dual-task portion of the adaptive gait test)	A trend toward a significant improvement in the BB group relative to the placebo (*p* = 0.065) and significant relationship between BB supplementation group and step error number under the dual-task condition t (*p* = 0.048)
Nilsson et al.^70^	Sweden	Healthy non-smoker volunteers, age: 50–70 years old, and BMI ≤ 28 kg/m^2^	1. Berry beverage (150 g blueberries, 50 g blackcurrant, 50 g elderberry, 50 g lingonberries, 50 g strawberry, and 100 g tomatoes/day) (n = 23) 2. control beverage(n = 23)	1. Berry beverage (150 g blueberries, 50 g blackcurrant, 50 g elderberry, 50 g lingonberries, 50 g strawberry, and 100 g tomatoes/day) (n = 20) 2. control beverage(n = 20)	Cross-over open-label RCT, 5 weeks	Verbal WM-test, SA test	Significantly better performance in WM test after the berry beverage compared to following the control beverage (*p* = 0.022)
Bowtell et al.^64^	England	Age > 65 years, ACEIII score of 88/100 and higher, and consuming less than five portions of fruit per day	1.Wild BB supplementation (30 mL\ concentrate providing 387 mg anthocyanidins) (n = 12) 2. Isoenergetic Placebo (n = 14)	1. Wild BB supplementation (30 mL\concentrate providing 387 mg anthocyanidins) (n = 12) 2. Isoenergetic Placebo (n = 14)	Parallel group double-blind RCT, 12 weeks	Brain perfusion, Task-related activation, Cognitive function (psychomotor function, visual processing, executive function, verbal and spatial memory, and WM)	Significantly better WM (2-back test) (*p* = 0.05), significant increases in brain activity within Brodmann areas 4/6/10/21/40/44/45, precuneus, anterior cingulate, and insula/thalamus (p < 0.001), and significant improvements in grey matter perfusion in the parietal (*p* = 0.013) and occipital (*p* = 0.031) lobes after blueberry vs. placebo supplementation
Whyte et al.^65^	England	Independently living healthy volunteers, age: 65–80 years old, all ethnicity, and subjective self-reported memory complaints	1.WBP (500 mg/ day) (n = 30) 2. WBP (1000 mg /day) (n = 31) 3.WBE (111 mg /day) (n = 31) 4.Placebo (n = 30)	1.WBP (500 mg/ day) (n = 28) 2. WBP (1000 mg /day) (n = 29) 3.WBE (111 mg /day) (n = 28) 4.Placebo (n = 27)	Parallel group double-blind RCT, 6 months	Verbal (RAVLT), visual (Corsi Block) and short term spatial episodic memory, WM, and executive function including selective attention	Significantly better performance in verbal episodic memory (*p* = 0.038), and a trend towards better performance in visual episodic memory following WBE111 intervention in comparison to placebo (*p* = 0.069)
Miller et al.^66^	USA	Age: 60–75 years, BMI: 18.5–29.9, adequate visual acuity, and > 12 months postmenopausal	1.freeze-dried BB (24 g/day) (n = 20) 2. Placebo (n = 20)	1.freeze-dried BB (24 g/day) (n = 19) 2. Placebo (n = 19)	Parallel group double-blind RCT, 90 Days	Executive function (TST, TMT), long-term memory (CVLT-II), short-term memory (DS task), spatial cognition (VMWM), and attention (ANT)	Significantly fewer repetition errors in the CVLT (*p* = 0.031) and reduced switch cost on a TST (*p* = 0.033) across study visits following freeze-dried BB intervention in comparison with placebo
Boespflug et al.^92^	USA	Age > 65 years, with mild cognitive impairment consistent with MCI	1. Whole freeze-dried BB powder (12.5 g per packet*2/day) (n = 11) 2. Placebo powder (n = 10)	1. Whole freeze-dried BB powder (12.5 g per packet*2/day) (n = 8) 2. Placebo powder (n = 8)	Parallel group double-blind RCT, 16 weeks	WM performance, and functional MRI	Increased BOLD activation in the left pre-central gyrus, left middle frontal gyrus, and left inferior parietal lobe during WM load conditions (p < 0.01) with no clear enhancement of WM following freeze-dried BB intervention in comparison with placebo
McNamara et al.^68^	USA	Age: 62–80 years old, and mild, self-perceived cognitive decline differentiated from MCI and prodromal AD	1. FO (400 mg EPA and 200 mg DHA × 4/day) + placebo powder (n = 21) 2. freeze dried BB powder (12.5 g*2/day) + placebo oil (n = 24) 3. FO + BB Powder (n = 26) 4. Placebo (n = 23)	1. FO (400 mg EPA and 200 mg DHA × 4/day) + placebo powder (n = 15) 2. freeze dried BB powder (12.5 g*2/day) + placebo oil (n = 16) 3. FO + BB Powder (n = 17) 4.Placebo (n = 17)	Parallel group double-blind RCT, 24 weeks	Psychomotor speed, WM, lexical access, new learning and long-term memory (HVLT), and cognitive symptoms in everyday activities (DEX)	Improved DEX following FO supplementation compared to placebo (*p* = 0.03) and following BB supplementation compared to placebo (*p* = 0.05) Improved HVLT for the BB-treated group compared to placebo (*p* = 0.04)
Bensalem et al.^63^	France & Canada	Age: 60–70 years old healthy subjects, BMI: 20–30, 26 < MMSE score ≤ 29 and usual dietary intake of red fruits ≤ 2 servings per week; tea consumption ≤ 1 cup per day; dark chocolate (≥ 70% cocoa) ≤ 140 g per week and less than 3 servings per week of omega-3 fatty acids rich foods such as fish, algae,…	1. 300 mg*2/day PEGB capsule (containing 258 mg flavonoids) (n = 101) 2. placebo (n = 105)	1. 300 mg*2/day PEGB capsule (containing 258 mg flavonoids) (n = 92) 2. placebo (n = 98)	Parallel group double-blind RCT, 6 months	PALTEA, VRM, and WM	Improved cognitive performances of participants with baseline PALTEA ≥ 57 errors, improved PALTEA (*p* = 0.037), VRMFR (*p* = 0.014) and the delayed version of the VRMR (*p* = 0.005) with no significant enhancement of WM following PEGB intervention in comparison with placebo
Dodd et al.^59^	UK	Age: 60–75 years, MMSE score: ≤ 25, and a depression index score on BSI : ≥ 11	1.a single dose flavonoid rich BB beverage (508 mg of antho- and 71 mg procyanidins) equivalent to approximately 200 g of fresh BB 2. Placebo beverage(n = 18)		Cross-over single-blind? RCT 2 h versus 5 h	Executive function and memory (Go-NoGo, Stroop, Digit Switch, Continuous Performance Task, Digit Symbol Substitution Test, Random Word Generation, Three-Word Sets Task, N-back, Letter memory, Location Task, immediate and delayed recall and recognition)	Significantly worse performance following the control drink at 2 h compared to 5 h post consumption (*p* = 0.04), better performance in the immediate word recognition task following the blueberry compared with the control intervention (*p* = 0.05), and significantly more word recognition following the blueberry compared to the control drink at the 2 h (*p* = 0.02)
Krikorian et al.^61^	USA	Age ≥ 68 years, with MCI confirmed	1.Whole BB powder packet contained 12 g powder *2/day (n = 24) 2. Placebo powder 10 g powder *2/day (n = 23)	1.Whole BB powder packet contained 12 g powder *2/day (n = 16) 2. Placebo powder 10 g powder *2/day (n = 21)	Parallel double-blind group RCT, 16 weeks	Speed of processing, WM, lexical access for semantic access, and verbal and nonverbal long-term memory	Improved semantic access s(* p* = 0.01) and better SPAL (*p* = 0.05) following the BB powder compared to the placebo powder

AD: Alzheimer's disease ‘AN: Attention Network Task, BB:blueberry, BMI: body mass index, BSI: the Brief Symptom Inventory, CVLT: the California Verbal Learning Task, CVLT-II: California Verbal Learning Test, 2nded, DEX: Dysexecutive questionnaire, DS: digit span, FO: fish oil; GDS: Geriatric Depression Scale, HVLT: Hopkins Verbal Learning Test, MCDR: modified Clinical Dementia Rating, MCI: mild cognitive impairment, MMSE: a Mini Mental State Examination, MoCA: Montreal Cognitive Assessment, PAL: Paired Associate Learning Test, PALTEA: PAL total errors adjusted, PEGB :polyphenol-rich extract from grape and blueberry, POMS: Profile of Mood States, RAVLT: Rey’s Auditory Verbal Learning task, RCT: randomized double-blind controlled trials, SA: Selective attention, TST: task-switching test, TMT: trail-making test, VMWM: virtual version of the Morris Water Maze, V-PAL: Verbal Paired Associate Learning Test, VRM: verbal episodic and recognition memory, WBE: Wild Blueberry extract, WBP: Wild Blueberry Powder, WM: Working memory.

### Cognitive function

#### Global cognitive performance

Dodd et al.^59^ in a cross-over RCT among 18 healthy older volunteers aged 60–75 years with MMSE score ≤ 25 reported significantly worse global cognitive performance subsequent the control drink between 2 and 5 h post-consumption, while there was no significant difference in global cognitive performance between 2 versus 5 h post-consumption of the blueberry beverage. This means that blueberries could be valuable in terms of preserving cognitive function in older adults. This research had a small sample size and the absence of statistical power may partially describe the non-significant findings in such a relatively heterogeneous sample.

#### Psychomotor function

Psychomotor performances comprise the combination of accurate motor reactions, attention, and problem-solving capabilities^60^.

Krikorian et al.^61^ detected a trend demonstrating better psychomotor processing speed (shorter time on task) in freeze-dried blueberry powder-treated older adults with MCI in comparison with placebo powder-treated subjects on the Trail- Making Test, part A after 16 weeks of supplementation. One of the main limitations of current trial was the relatively small sample size. Although the findings have been inspiring, there is an obvious necessity for larger trials.

Schrager et al.^62^ studied whether six weeks of daily intake of two cups of frozen blueberry would affect older adults (n = 20). They found no significant variations in either the blueberry or placebo groups in simple reaction time with supplementation. Additionally blueberry intake resulted in no significant changes in gait speed under the single-task condition. Substantial within-group improvement in single-task step errors was established in the blueberry group. After adjusting for gait speed, the trend toward a significant improvement in the blueberry group diminished compared to the placebo group in the single-task condition. This study had a small and heterogeneous sample which probably resulted in non-significant results.

#### Learning and memory

In the RCT by Bensalem et al.^63^ 6-month supplementation with polyphenol-rich extract from grape and blueberry (PEGB) led to the higher total number of accurate words at the immediate recall in comparison with the placebo among 60–70-year-old healthy subjects with a BMI between 20–30 and 26 < MMSE score ≤ 29. Additionally, performance at the verbal episodic and recognition memory-free recall (VRMFR) was revealed to be affected by PEGB. Furthermore, PEGB led to lower paired associates learning total errors adjusted (PALTEA) among healthy elderly participants in comparison with the baseline scores. A significant interaction was observed between the PEGB effect and the baseline PALTEA score. However, no significant difference was seen between PEGB and placebo groups at the end of the intervention across the entire trial population. PEGB supplementation was established to be considerably more efficient in enhancing cognitive performances among subjects with ≥ 57 errors in the baseline PALTEA (subjects with the highest cognitive impairments). A high variability in baseline PAL score was observed in this study. Furthermore, the people in the control group had a lower PALTEA score at the second appointment than at baseline, probably because of a test–retest effect. This effect would reduce the likelihood to observe a substantial between-group difference, so the PEGB effects presented in this study may have been underestimated.

In the study of Dodd et al.^59^ intake of a single dose of blueberry beverage resulted in better performance in the immediate word recognition task compared to the control intervention. In spite of no substantial intervention by time interaction, significantly more words were recognized 2 h after the blueberry consumption compared to the control drink. However, intake of a single dose of blueberry beverage had no beneficiary effect on delayed word recognition task compared to the control intervention. The small sample size may describe the non-significant results of this study.

Bowtell et al.^64^ showed that 12 weeks of daily intake of blueberry concentrate supplementation improved international shopping list task with no significant difference compared to the placebo among 26 healthy older adults. The primary limitation of this study was the small sample size.

Krikorian et al.^53^ examined the efficacy of daily drinking of wild blueberry extract for 12 weeks in a sample of nine older adults with early memory changes. Blueberry juice significantly enhanced the word list recall and paired associate learning. The discoveries of this trial propose that moderate-term supplementation with blueberry can provide a neurocognitive advantage.

The small sample size and lack of a completely matched control product were the primary limitations of this study. However, the relatively greater glycemic content of the grape juice as the placebo beverage may have influenced the cognitive performance.

The word recognition procedure wants the participants to identify previously presented words from the main word list whereas ignoring words from another word list and further phonetically or semantically matched diverse words. It has been known that episodic memory performance would decrease in older people^65^.

In a double-blinded, placebo-controlled RCT by Whyte et al.^65^ daily supplementation with purified wild blueberry extract at 100 mg (WBE111) for six months resulted in a significantly improved function in late word recognition in the Reys Auditory Verbal Learning Task (RAVLT) (in contrast with the placebo in 65–80-year-old healthy subjects with self-reported memory complaints). These impacts were not seen for whole wild blueberry powder at 500 mg (WBP500) and 1000 mg (WBP1000). Furthermore, daily WBE111 supplementation caused an improving trend in visuospatial Corsi Block function (the entire number of orders properly recalled) in contrast with the placebo after 3 months of supplementation.

Miller et al.^66^ showed that 3-month supplementation of 37 men and women (age 60–75 years) with MMSE score ≥ 24 with freeze-dried blueberry resulted in fewer repetition faults according to CVLT, 2nd Ed.^67^ after the end of intervention than they did at the baseline.

In the RCT by Krikorian et al.^61^ freeze-dried blueberry fruit powder supplementation resulted in a significantly better nonverbal memory performance (SPAL) compared to the placebo powder in adults aged 68 years and older with MCI. In spite of the obvious improvement in the Hopkins Verbal Learning Test (HVLT) recall favoring the blueberry group, it was not a significant effect. An effect was observed also for semantic access and not for phonological access favoring the blueberry-supplemented group. The relatively low sample size may partially describe the non-significant findings in this sample.

McNamara et al.^68^ examined the effect of 24-week supplementation with daily fish oil (EPA and DHA) or blueberry or both in elderly men and women aged 62–80 years old with mild, self-perceived cognitive deterioration in a randomized, double- blind, placebo-controlled trial. This was monitored for a further 24 weeks. There was an effect demonstrating enhanced insight in recognition memory on the HVLT for the blueberry-treated group. The memory insight improvement in the blueberry group was not preserved at week 48 (24 weeks following cessation of the supplementation).

#### Working memory (WM) capacity

WM represents the limited quantity of info that can be kept in mind and used in the practical implementation of cognitive jobs. It has often been correlated to intelligence, data processing, executive function, understanding, problem-solving, and learning^69^.

Nilsson et al.^70^ in a randomized crossover trial showed that supplementation for five weeks among 46 healthy men and women within 50–70 years of age with a berry drink based on a combination of berries (blueberries, blackcurrant, elderberry, lingonberries, strawberry, and tomatoes) enhanced the performance significantly in the WM-test at 30 min by nearly 5% in contrast to the control beverage. The test employed in the current study demonstrated an extension of the Radeborg et al. developed methodology^71^. The tests consisted of twelve sets of short indicative sentences that could be either meaningful ‘the boy came back from school’, or absurd, such as ‘the cat struck the concept’. The sentences were read to the participants, and instantly after each sentence the participant had to specify if the sentence was meaningful or not. After each set of sentences, the participants had to repeat the first noun in each of the sentences. An apparent limitation of this study was that it was not probable to blind the products to the participants because of the evident dissimilarities. Furthermore, the test product included a mix of berries which discard possibilities to provide any suggestion concerning individual berries.

Krikorian et al.^61^ found no effect for task switching as measured by the Trail-Making Test, part B task following blueberry supplementation in older adults with MCI compared to the placebo. No notable effect of PEGB was observed either by Bensalem et al.^63^ for the WM measured through the Spatial Span (SSP) and the Reverse SSP.

In an RCT by Whyte et al.^65^ daily supplementation with WBP or WBE for six months was not associated with encouraging impacts in older adults. WM was assessed through implementation of two different tasks, involving serial subtractions as designated beforehand by Bell et al.^72^ plus Sternberg memory scanning. Probably, an element of practice effect occurred whereby, participants enhanced their approach on performance in these memory tasks thus decreasing the task sensitivity to the treatment. Additionally, possible deterioration of the capsule active constituents at 6 months and increased tolerance to the WBE are other issues to be considered.

#### Executive function

Miller et al.^66^ reported that 3-month supplementation of 37 men and women (ages 60–75 years) with MMSE score ≥ 24 with freeze-dried blueberry resulted in a greater reduction in switch stimuli errors between follow-ups in task-switching test (TST) [a practice visit (visit 1), a baseline visit (visit 2), and 45- and 90-day interposition visits (visits 3 and 4, correspondingly)] in comparison with the control. When group differences in physical activity and computer use were measured, the interposition endured. Post hoc analysis showed that, within the blueberry group, performance on the baseline and 90-day intervention visits as well as 45- and 90-day interposition visits were meaningfully different even when the Bonferroni modification was exerted.

In the study by Dodd et al.^59^ a tendency towards lower switch cost was detected following the blueberry usage in comparison with the control beverage at the 2-h time point; however, the overall intervention by time interaction proved insignificant.

Bowtell et al.^64^ showed that 12 weeks of the daily intake of a blueberry concentrate in healthy older adults improved WM and also the Groton maze learning role in comparison with the placebo. The WM was assessed by means of an n-back task, including 1-back and 2-back memory task blocks. Although the trial did not report significant effects in cognitive function, the percentage change in presentation of the 2-back tests showed a piece of weak evidence for upgrading in the blueberry versus the placebo. Although the findings are encouraging, there is a clear need for larger trials.

Krikorian et al.^61^ observed no effect for perceived cognitive efficiency in daily tasks as measured by the dysexecutive questionnaire (DEX) after 16 weeks of supplementation with blueberry powder in comparison with the placebo.

Daily consumption of two cups of frozen blueberry for 6 weeks resulted in a significant improvement in foot placement and balance control during computerized dual-task testing of gait compared to the placebo in the Schrager et al.’s study^62^.

In the Whyte et al.’s study^65^, daily supplementation with WBE or WBP was correlated with no promising impacts on executive function and attention measured by modified attention network task (MANT) as described by Whyte et al.^73^ Meanwhile, there were only two blocks with a stimulus length of 500 ms, with no noise condition applied.

#### Brain perfusion/activity

Bowtell et al.^64^ showed that 12 weeks of the daily intake of a blueberry concentrate in healthy older adults resulted in significant improvements in brain activity in response to blueberry supplementation compared to the placebo group within Brodmann zones 4/6/10/21/40/44/45, precuneus, anterior cingulate cortex (ACC), and insula/thalamus, along with significant enhancements in grey matter perfusion in the parietal plus occipital lobes.

Brodmann area 21 [middle temporal gyrus (MTG)] is implicated in sound recognition as well as word and picture semantic processing^74^, language processing^75^, decoding gaze direction, deductive reasoning^76^ plus intelligible speech and processing of verbal mental arithmetic^77^. Brodmann area 40 (supramarginal gyrus) plays a role in visual word recognition, auditory memory processing, and emotion recognition^78–80^. The left-hemisphere Brodmann areas 44 and 45 are associated with language processing (including production and comprehension)^81^ plus higher cognitive functions including music, calculus, and WM^82^. The area 44 is involved in conditions of vicarious learning, i.e., attentive consideration of activities of another^83^. ACC plays a substantial role in attention, performance monitoring, error processing, emotional information processing, and motivational behavior^84–88^. Improved ACC activity is related to faster speed in a reaction time task^89^. The insula has various roles in humans from sensory and affective processing to high-level cognition (attention plus salience processing and speech)^90^. The thalamus is an important node in linkages enduring cognitive performances, identified to deteriorate in usual aging, comprising procedures of memory as well as executive functions and information processing^91^.

Boespflug et al.^92^ in a double-blind, placebo-controlled RCT assessed the effect of daily blueberry supplementation or placebo powder for 16 weeks on blood oxygen level-dependent (BOLD) signal in sixteen 68-year and older adults with MCI. Blueberry-treated patients displayed augmented BOLD activation in the left pre-central gyrus, left middle frontal gyrus (LMFG), and left inferior parietal lobe throughout working memory load situations. A rising number of investigations have studied how motivation interrelates with particular cognitive performances, comprising attention, WM, and other executive functions. It’s true for a subset of brain regions, which included the left pre-central gyrus^93^. The LMFG plays a crucial role in the progress of executive attention, literacy, and word production^94–96^. Left inferior parietal lobe is implicated in language comprehension plus production and converges semantic information pathways^97,98^.

## Discussion

To our knowledge, this is the first systematic review of available clinical trials on the effects of berry-based supplements and foods on cognitive performances and brain perfusion parameters in elderlies with normal cognition or MCI. This review of 11 clinical trials provided inconsistent findings across the measured outcomes. Given the heterogeneity of study design, intervention type/dose/duration, outcomes of interest and cognitive tasks used, the described trials have revealed that berry-based supplements and foods may have beneficial effects on global cognitive performance (one study)^59^, psychomotor function (two studies)^61,62^, learning and memory (nine studies)^53,59,61,63–66,68^, WM capacity (three studies)^61,65,70^,executive function (six studies)^59,61,62,64–66^, and brain perfusion/activity (two studies)^64,92^. Nine of 11 trials were of poor or modest methodological quality principally because of suspicions about the blinding of outcome assessment, allocation concealment and random allocation to groups. The exact interventions given in the included trials were heterogeneous and in some trials poorly explained.

Fruit juice and powder may be impressive approaches to enhancing the total fruit intake. Regarding the nutritional worth, freeze-dried powder that is free of water maintains concentrated bio-available antioxidants, fiber, and other ingredients^99^. Some studies have proposed that the juicing procedure can result in a poor content of fiber and some bio-actives comprising polyphenols, vitamins, and minerals^100,101^, while another study suggests that processing can improve the carotenoid bioavailability^102^. On the other hand, the microstructure of the extract in contrast to the powders must also be considered. The extract lacks fibers and is water-soluble, while the full spectrum powders are very rich in insoluble fibers to which the polyphenols are bound. It is thus likely to assume that the polyphenols in the powders would not be as bioavailable as in the extract, and may hence be less worthwhile.

Most included studies had small sample sizes which may explain the non-significant findings. In one study, the product involved a mix of berries which made it impossible to give any conclusion regarding individual berries. In studies with longer duration of treatment, possible deterioration of the capsule’s active constituents and increased tolerance to the product are other concerns. In most included studies, beneficial properties were established with dosage, which is achievable for daily consumption. Although other studies used higher amounts, this may only be possible for a limited therapeutic period. Nonetheless, establishing the involvement of berry metabolites in the cognition is puzzling, chiefly because berries have a different phytochemical profile, which can be extremely different across varieties.

Berries can be found as various little red, purple, or blue fruits. The frequently consumed berries include the blackberries (*Rubus *spp.), blueberries (*Vaccinium corymbosum*), cloudberry (*Rubus chamaemorus *L.), cranberry (*Vaccinium macrocarpon*), red raspberries (*Rubus idaeus*), black raspberries (*Rubus occidentalis *L.), and strawberries (*Fragaria x ananassa*). Less frequently consumed berries are black currants (*Ribes nigrum*), chokeberries (*Aronia melanocarpa*), lingonberry (*Vaccinium vitis-idaea *L.) and mulberries (*Morus alba *L.). Berries are eaten both as fresh fruit in addition to processed food products (for example beverages, extracts, jams, jellies, and freeze-dried). Generally, berries have low calories and high fiber, and contain different natural antioxidants such as vitamins C and E, plus other nutrients such as folic acid, calcium, selenium, alpha and beta carotene, and lutein. Berries are rich in polyphenols, with significant amounts of flavonoids (anthocyanins, flavonols, and flavanols), condensate tannins (proanthocyanidins), hydrolyzable tannins (ellagitannins and gallotannins), phenolic acids (hydroxybenzoic and hydroxycinnamic acids, chlorogenic acid), stilbenoids, and lignans^103,104^. Anthocyanins are a major group of natural, water-soluble pigments and provide the bright appearance to berries. Around 400 anthocyanins have been distinguished, which are mostly concentrated in the fruit external layer, particularly berries. Red berries, strawberries, and cherries have also anthocyanins in their body^105^.

When findings of the dietary intermediations systematically reviewed in this study are weighed together, it seems that there are numerous essential mechanisms whereby berries may potentially provide cognitive health properties. The brain is predominantly exposed to neuro-inflammation and oxidative stress. This susceptibility further grows with age^70^. Amplified reactive oxygen species (ROS) production or reduced antioxidant protection is described as oxidative stress, which may contribute to the increase in various disorders such as neurodegenerative diseases^106^. Several medicinal properties of berries against oxidative stress-related disorders have been associated with their great content of phenolic antioxidants, particularly anthocyanin and phenolic acids. The antioxidant effects of anthocyanins were evaluated in vitro, comprising 1,1-diphenyl-2-picrylhydrazyl, radical scavenging property, oxygen radical absorbance capability and ferric decreasing antioxidant potential^107–109^. Furthermore, berries have proved to contain an acceptable content of vitamin A, vitamin, C and vitamin E, which function as antioxidants^31^. On the other hand, it has been discussed that inflammatory cytokines in the circulation can cross the blood–brain barrier (BBB), initiate a neuro-inflammatory condition, disturb neuroendocrine performance as well as neurotransmitter arrangements, finally resulting in cognitive impairment^110^. The transcription factor nuclear factor kappa-light-chain-enhancer of activated B cells (NF-κB) signaling pathway has a critical role in organizing inflammatory reactions^111^. Anthocyanin shuts off the initiation of NF-κB and activation of nitric oxide synthase-2, cyclooxygenase-2, interleukin-1β (IL-1β), and tumor necrosis factor-α (TNF-α) in macrophages^112,113^. Furthermore, anthocyanins are correlated with down-regulation of inflammatory cytokine genes including interleukin-6 and monocyte chemo-attractant protein-1 in adipose tissue^114^. Berry polyphenols improve mitochondrial function in intestinal Caco-2/15 cells activated with lipopolysaccharide (LPS), which reduces intestinal inflammation^115^. Metabolites of anthocyanin imitate active agents such as the anti-inflammatory salicylic acid (2-hydroxybenzoic acid) and are associated with useful variations in biomarkers of inflammation in in-vitro models^35,116^. A rodent model also revealed that anthocyanin intervention suppressed the biological activity of cyclooxygenase-2^117^. So anthocyanins decrease the omega-6 eicosanoids turnover (for example, prostaglandin E2 (PGE2) and leukotriene B4 (LTB4)). Additionally, anthocyanin microbial metabolites, such as 3,4-dihydroxybenzoic acid (protocatechuic acid), 4-hydroxy-3-methoxybenzoic acid (vanillic acid), 4-hydroxy-3,5-dimethoxybenzoic acid (syringic acid), and 3,4,5-trihydroxybenzoic acid (gallic acid), are related with advantageous variations in biomarkers of inflammation in in-vitro models^118^.

Meanwhile, phenolic compounds are shown to be extensively metabolized into simple phenolic metabolites through the action of microorganisms in the colon^119^. These compounds may modify gut microbiota by stimulating favorable bacteria and deterring pathogenic bacteria. In healthy individuals, the gut microbiota modulations largely result in an increase in Bifidobacterium, Lactobacillus and Akkermansia, thus proposing a prebiotic-like property of the berries or their compounds^119–122^. Epidemiological explanations and studies in animal models confirm a common schema that involves the gut microbiota over the microbiome-gut-brain axis in the pathogenesis of neurodegenerative diseases such as dementia^123^.

The current study had some limitations that should be considered in explaining the outcomes of this systematic review. First, despite the collective body of nutraceutical studies, the number of studies included in this explicit review after a systematic review of the medical literature was less than what would have been expected. Secondly, there are two aspects including possible publication bias and the selected search terms that could have influenced the results of the review. Some unpublished abstracts and articles have not been included due to their unavailability. Further, we nominated only the English and Persian language due to limited resources, which in turn would increase bias. These may significantly reduce the size of our sample and therefore our capacity to explain statistically important findings. Another weakness of this review was due to the heterogeneity of the selected studies which considered a variety of outcome events. The confounding effects of variations in background polyphenol intake among the studies must be considered in interpreting the results. As a final point, there may be some probable parts not considered in the current systematic review, such as the magnitude of IQ, region, diet, and race. Furthermore, this review relies on the findings of available published studies, so any limitations of these studies are unavoidable limitations of our review study as well. Despite the specified limitations, this systematic review has been the first systematic review of clinical trials investigating the effect of berry-based dietary interventions on cognitive function.

In included trials, the highest duration of berry-based food or supplement administration was 6 months. Furthermore, most studies had relatively low sample sizes. Future investigations should attempt to clarify the potential special effects of berry-based food or supplement consumption in a prolonged period of treatment to distinguish possible significant enhancements in cognition. Furthermore, the safety of these interventions should be described by means of a systematic method of recording probable adverse events based on a good clinical practice.

This review intended to collect and discuss scientific evidence concerning the beneficial role of consumption of berry-based products on the prevention of cognitive decline. In summary, from available human studies, intake of whole berries or berry-based products, principally consumed as fresh or frozen fruit, or as unsweetened beverages, may be endorsed as part of a healthy dietary approach to preventing cognitive decline among the elderly with healthy cognition or MCI. The future well-controlled dietary intervention-based investigations, preferably clinical trials, should more carefully choose appropriate supplements (as crude materials or whole berries) with different dosing regimens to determine the accurate dose. Individualized dietary and nutritional recommendations, definitely through metabotypes determination, seem to be an achievable approach for taking full advantage of any benefits provided by berries and their polyphenols.
